# Epidemiological of chronic kidney disease based on a database of health

**DOI:** 10.1590/1806-9282.20240644

**Published:** 2024-12-02

**Authors:** Bertrand Saulo Vieira Cariry, Ysabely de Aguiar Pontes Pamplona, Fernando Luiz Affonso Fonseca, Lourdes Conceição Martins

**Affiliations:** 1 Mestre Vitalino Hospital – Caruaru (PE), Brazil.; 2 Universidade Católica de Santos – Santos (SP), Brazil.; 3 Centro Universitário Faculdade de Medicina do ABC – Santo André (SP), Brazil.; 4 Universidade Federal de São Paulo – Diadema (SP), Brazil.

**Keywords:** Chronic kidney disease, Dialysis, Epidemiology, Database, Public health

## Abstract

**INTRODUCTION:**

Chronic kidney disease has presented frequencies that exceed the installed capacity of dialysis services, and data on prevalence are uncertain worldwide.

**OBJECTIVE:**

The objective of this study was to analyze the epidemiological profile of patients assisted in renal replacement therapy and details on the assistance provided.

**METHODS:**

Population-based cross-sectional study with information obtained from the Unified Health System database. The research location was the state of Paraíba, and the observation period was from January 1, 2009, to December 31, 2013, n = 64,676 documents, and the variables observed were: gender; age group, race, or color; place of residence; place where the therapy was performed; vascular access; modality if hemodialysisor peritoneal dialysis; registration on the kidney transplantation list; infection by hepatitis B, C, and HIV viruses; and costs.

**RESULTS:**

A higher prevalence of request for highly complex procedure was observed in males (56.8%) and white (39%). The cities with the highest prevalence of dialysis patients were João Pessoa, Campina Grande, Santa Rita, Sousa, Bayeux, and Patos. Most of the sample referred to patients undergoing hemodialysis as a renal replacement therapy modality, with 42.2% not having definitive arteriovenous fistula access. The renal replacement therapy expenses observed were increasing, and in 2009, they accounted for 2.89% of health spending, reaching 3.32% of state health spending in 2013.

**CONCLUSION:**

Using secondary data from DATASUS to understand the population with chronic kidney disease and the assistance offered is possible.

## INTRODUCTION

Chronic kidney disease (CKD) is defined as an abnormality of kidney structure or function present for more than 3 months with health implications, classified according to cause, glomerular filtration rate into stages E1a to E5, and the degree of albuminuria into stages A1 to A3. It is a serious illness characterized by a gradual and insidious loss of kidney function and the need for provision of renal replacement therapy (RRT) for life maintenance, which includes peritoneal dialysis (PD), hemodialysis (HD), and kidney transplantation (KT). Common risk factors for CKD include systemic arterial hypertension, diabetes mellitus, cardiovascular disease (CVD), and a previous history of acute kidney injury. Other factors associated with CKD development, as cited in the KDIGO (KidneyDisease: Improving Global Outcomes) review 2023, include genitourinary disorders with structural alterations; recurrent nephrolithiasis; systemic diseases affecting multiple organs such as systemic lupus erythematosus, gout, and HIV infection; occupational exposure to heavy metals such as cadmium and mercury, or polycyclic hydrocarbons and pesticides; family history of CKD, whether or not genetic abnormalities are identified; and gestational conditions such as preterm birth, low birth weight, with outcomes for the fetus, and preeclampsia and eclampsia as risk factors for the mother^1,2^.

Data on the global prevalence of CKD are uncertain, varying in regions worldwide, with this variation being strongly related to the lack of local records of the disease occurrence. CKD requires a differentiated approach to care, where symptom burden reduction, social and functional rehabilitation, and relief of suffering for those affected may be priorities rather than reducing mortality and increasing longevity. Another peculiar aspect of this nosology is its cost implication, as CKD progression increases the costs of patient therapeutic management. In advanced stages of the disease, then termed end-stage renal disease (ESRD), patients will require RRT for life maintenance, which currently has a worldwide demand growth rate of 7% per year, exceeding the installed capacity of RRT services and funding sources in developing countries such as Brazil^3,4^.

Therefore, the aim of this study was to demonstrate the epidemiological profile of incident and prevalent CKD patients in RRT, as well as to detail the care provided based on information such as the modality of RRT, type of access for RRT, whether catheter or arteriovenous fistula (AVF), frequency of chronic viral co-infections and CKD/RRT, as well as clinical fitness and access to KT.

## METHODOLOGY

This is a cross-sectional observational quantitative study of the population-based type, and information used was extracted from secondary databases of the Ministry of Health regarding RRT, which is an integral part of the APAC (request for highly complex procedure) subsystem, called APAC/RRT, being pointed out as a rich repository of data on RRT and its patients in Brazil, due to the wealth of epidemiological information available and the possibility of monitoring historical series it allows. It differs from other health information systems in the country by the degree of detail of the records. The information managed by it, in addition to being important for understanding the epidemiological profile of the patients attended, allows the assessment of care based on management indicators, as well as the monitoring and surveillance of CKD and RRT funded by the Unified Health System (UHS). The data are public, anonymized, and in compliance with the requirements of the Access to Information Act. The research site chosen was the state of Paraiba (PB), and RRT data for the period from January 1, 2009, to December 31, 2013, totaling 5 years of observation, were analyzed. All APACs referring to prevalent patients in RRT funded by Unified Health System (UHS) for the period were included. The created database gathered information on: prevalent patients in some form of RRT, and descriptive socio-demographic variables (gender, color or race, age, and municipality/region of residence); treatment modality (HD and PD); treatment time; clinical service where RRT is performed; information on costs and service providers; reasons for treatment exit, if discharge; conservative treatment; transfers between RRT services; frequency of deaths; diagnosis of diseases associated with CKD, coded according to the International Statistical Classification of Diseases and Related Health Problems-Tenth Revision; user mortality; type of access used for RRT whether AVF or double-lumen catheter (DLC); weight and height of users; patient travel to clinics offering RRT, observed from the place of residence and treatment location; frequency of patients with hepatitis B, hepatitis C, and HIV infection in RRT; presence of residual diuresis; blood sugar; and albumin and hemoglobin of patients assisted^5^.

The number of APAC reports studied was 64,676 digitized reports. Each APAC records a portion of the patient's treatment history, like a patient who undergoes multiple hospitalizations in a hospital setting, but each encounter is considered a new case, as RRT occurs on an outpatient basis, with patients undergoing a 4-h HD session at the clinic or performing PD sessions at home. Initially, a descriptive analysis of all study variables was conducted. Qualitative variables were presented in terms of their absolute and relative values, and quantitative variables in terms of their central tendency and dispersion values. The chi-square test was used to assess the association between qualitative variables. In all models, a significance level of 0.05 was adopted. The Statistical Package of Social Science for Windows, version 17, was used for analysis.

The study was submitted for approval by the Research Ethics Committee of the Faculty of Medicine and Nursing Nova Esperança, through the Plataforma Brazil, and was reviewed and approved in accordance with Resolution of the National Health Council no. 466, 2012, which regulates research involving human subjects, and in compliance with the norms of the national Medical Code of Ethics, Chapter XXII, which deals with medical research. The transformation of administrative databases into research databases was carried out with a guarantee of confidentiality and anonymity of the research subjects. The risks of the research included a breach of the anonymity for the users involved in the study, which was not possible given the significant expected benefits, with the possibility of using information for health decision-making while respecting social, cultural, ethical, moral, and religious values, as well as the habits and customs of the community.

## RESULTS

In the analysis of the gender, it was coded as "M" or "F," excluding any other values. It was observed that there was a predominance of reports for male (M) patients during the period, accounting for 57% of the sample, while 43% were for female (F) patients. Regarding race or ethnicity, it was observed that 39.9% were classified as brown, 38.2% as white, 2.8% as black, 0.1% as yellow, and only two APAC reports were for indigenous patients. Information on this variable was not available for 19% of the APAC reports.

Most APAC reports were for patients aged between 20 and 25 years and 60 and 64 years, comprising 86.91% of the total sample, corresponding to the usual working age in the country. Regarding elderly patients in RRT over 65 years, it was observed that 13.69% of the frequencies were for this population of patients receiving RRT funded by UHS.

The annual frequency of APAC reports increased from 2009 to 2013 in all regions, with the João Pessoa region gathering the highest frequencies of reports, totaling 4,915 in 2009 and reaching 5,880 for the year 2013. Regarding the RRT modality, HD predominated in all three regions, with the following frequency distributions: 83.7% in João Pessoa, 99.4% in Campina Grande, and 98.2% in Sertão. João Pessoa is also the region that had the highest frequency of APAC reports for patients on PD modality, accounting for 16.3% of the total sample.

The municipalities in PB with the highest number of patients using RRT services, according to the sample data, were, in order of highest frequency: João Pessoa with 10,327 APAC reports (20.5%); Campina Grande with 9,837 APAC reports (19.5%); Santa Rita with 2,130 APAC reports (4.2%); Sousa with 1,488 APAC reports (3%); Bayeux with 1,433 APAC reports (2.8%); Patos with 1,157 APAC reports (2.3%); Queimadas with 970 APAC reports (1.9%); Mamanguape with 684 APAC reports (1.4%); Cabedelo with 607 APAC reports (1.2%); Pedras de Fogo with 546 APAC reports (1.1%); Pombal with 503 APAC reports (1%); and Sape with 497 APAC reports (1%). All other municipalities in the state had frequencies for RRT/nephrology services of less than 1% of the total sample. Frequencies were presented in the form of maps showing the distribution of frequencies by postal address code (ZIP) of patients’ residences, as shown.

An analysis of APAC frequencies by type of access for RRT, categorized as AVF or non-AVF, revealed that the use of AVF was frequent in 56.3% of the sample, while 42.2% of the sample were on RRT with DLC or were on PD modality. It was observed that most patients in João Pessoa underwent RRT using AVF, accounting for 78.5% of the sample, while the frequency of reports for patients on RRT using other access, not AVF, predominated in Campina Grande (53.6%) and in Sertão (63.1%) (Table 1).

**Table 1:** Distribution of APAC frequencies by region of Paraiba, year, and race or color.

		João Pessoa		Campina Grande		Sertão		Significance level&
		n	%	n	%	n	%	
Year								
	2009	4,951	18.3	4,183	16.1	2,536	21.6	<0.001
	2010	5,311	19.7	4,635	17.9	2,395	20.4	
	2011	5,371	19.9	5,013	19.3	2,404	20.4	
	2012	5,481	20.3	5,742	22.2	2,409	20.5	
	2013	5,880	21.8	6,348	24.5	2,017	17.1	
Color or race								
	White	5,478	20.3	8,620	31.9	10,988	93.4	<0.001
	Black	1,320	4.9	401	1.5	73	0.6	
	Brown	18,446	68.3	6,835	26.4	533	4.5	
	Yellow	3	0	8	0	48	0.4	
	Indigenous	2	0	0	0	0	0	
	Without information	1,745	6.5	10,417	40.2	119	1.0	
Modality of RRT								
	Hemodialysis	22,590	83.7	25,774	99.4	11,552	98.2	<0.001
	Peritoneal dialyses	4,404	16.3	147	0.6	209	1.8	

**Figure 1:**
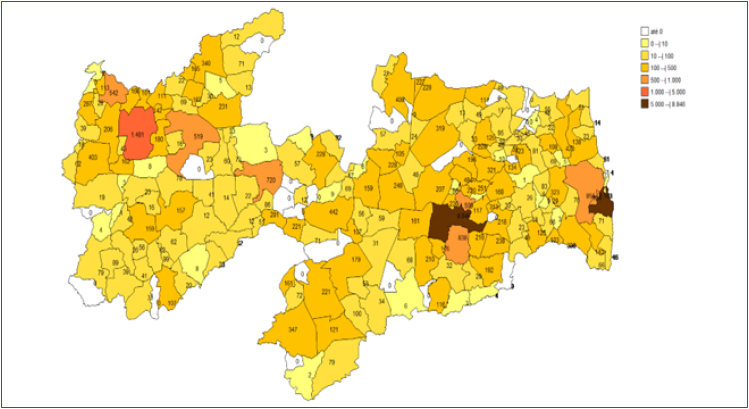
Frequence map of distribution of APAC for municipalities of residence.

& Chi-square test. The distribution of reports by region of the state was carried out, categorized into João Pessoa, Campina Grande, and Sertão, and it was based on the observation of treatment locations, using the identification numbers of the services provided by the National Registry of Health Establishments (CNES). The frequencies of all variables were compiled annually (Figure 1). RRT: renal replacement therapy.

About KT analyzed by region, it was observed that 57.4% of patients from João Pessoa, 48.5% from Campina Grande, and 61.7% from Sertão were on the waiting list for KT.

In the study sample, it was also possible to identify the prevalence of patients in RRT with associated infections by hepatitis C, hepatitis B, and HIV. The identification of these patient cohorts separately has clinical and managerial implications since the reuse of dialysis capillaries is still a practice in Brazil. A frequency of 2% of APAC reports was observed for patients with hepatitis C, and 0.2% for APAC reports for patients with hepatitis.

To comprehend the magnitude of expenditures on RRT within the UHS budget, a survey of total healthcare expenses per year was conducted using the Public Health Budget Information System (CNES) of the Health Ministry. The percentage of commitment to RRT was then calculated in relation to total healthcare spending for the state per year. The results found were as follows: in 2009, RRT accounted for 2.89% of healthcare spending in Paraiba; in 2010, this percentage was 2.97%; in 2011, RRT expenditure was 3.08% of total expenses; in 2012, it corresponded to 2.7% of the total; and by 2013, expenditure on RRT accounted for 3.32% of the state's health budget.

## DISCUSSION

According to the data, as APAC data is anonymized, it is not possible to identify the total number of dialysis patients. There was an observed increase in demand for RRT over the 5-year observation period, with a 3.5% increase in demand over 5 years, and each successive year contributed a greater amount to the total percentage of APAC. The frequency varied from 11,670 APAC in 2009 to 14,245 APAC in 2013, revealing an increase in demand for RRT over the period. These data are in line with information from the International Society of Nephrology (ISN), which describes that the prevalence of patients with HD is increasing rapidly in Latin America at a rate of 4% per year, while in Europe and the USA, the rate is growing at 2% per year^4^.

With a global population of 4 million people, 140,000 of these in Brazil, dependent on RRT for survival, patients still present high frequencies of morbidity and mortality. According to data from the Brazilian Society of Nephrology (SBN), in 2019, the annual national mortality rate was 18.2% of the total population, with the UHS covering 79% of the costs of RRT in Brazil, and in 2011, we had 643 dialysis units aiding with patients with CKD. Around 10% of these centers were in the Northeast region of Brazil, and, according to the analysis, 1.5% were in PB, with 84% of RRT carried out for the period being at the national level, according to SBN, funded by UHS. At the time, 10 services providing RRT were identified for the state, already described above, being located, 40% of them in the capital, with 60% of the frequencies of patients performing RRT in the interior, Campina Grande accounting for the same 40% of the sample, and 20% for the hinterland. In 2011, the Brazilian population was 198.2 million inhabitants, and for the SBN there was a record of 91,314 patients in RRT, highlighting that in 2000 the population in RRT was 42,629 patients, demonstrating a 2.14-fold increase in the frequency of patients on RRT, for Brazil, over a period of 11 years, and for PB, an increase of 1.18 times in 5 years^6,7^.

Although AVFs are ideal accesses for HD, the target of over 90% of accesses being AVFs was awarded by the National Kidney Foundation, in the USA, and by the European Guidelines for Best Practices in Dialysis, a seal of quality for the RRT, and it was not achieved in any region of the state, especially in the interior of the state, where the frequency of AVF accesses was lower^8^ (Table 2).

**Table 2:** Distribution of APAC frequencies for waiting list for renal transplant, type of access for renal replacement therapy, and positive serology for hepatitis C, B, and HIV for the region of Paraiba.

			João Pessoa		Campina Grande		Sertão		Significance level&
			N	%	n	%	n	%	
Waiting list for TX									
		Yes	13,517	57.4	10,005	48.5	5,685	61.7	<0.001
		No	10,044	42.6	10,627	51.5	3,526	38.3	
Type of access to RRT									
		AVF	20,719	78.5	11,361	44.4	4,319	36.9	<0.001
		No AVF	5,671	21.5	14,204	55.6	7,401	63.1	
Serologies positives									
		Hepatitis C							
		Positive	539	2.0	154	0.6	72	0.6	<0.001
		Negative	26,455	98.0	25,767	99.4	11,689	98.8	
		Hepatitis B							
		Positive	50	0.2	34	0.1	34	0.3	<0.001
		Negative	26,944	99.8	25,887	99.9	11,727	99.7	
		HIV							
		Positive	108	0.4	1	0	0	0	<0.001
		Negative	26,886	99.6	25,920	100	11,761	100	

& Chi-square test. TX: kidney transplantation; RRT: renal replacement therapy; AVF: arteriovenous fistula.

The recording of data on RRT plays a fundamental role in evaluating the results and quality of services offered. The existence of a sufficiently large health information registry, capable of measuring and reporting dialysis care indicators, is a crucial step in understanding the RRT provided. The absence of these records implies barriers: for the patient, access to equitable and highly efficient care; for the manager, the possibility of developing effective strategies for risk reduction. The main indicators suggested for observation are vascular access problems; dialysis adequacy; fatigue; CVD; mortality; in addition to observing other variables involved in care provision, such as the quality of communication and standardization of health records^9,10^.

Current trends are worrying, and from a global perspective, costs are unsustainable even for developed countries, and there is a significant worldwide refusal of treatment by patients who develop CKD and renounce treatment, resulting in millions of avoidable deaths every year. The projection for 2030 is that there will be about 14.5 million people with CKD progressing to ESRD, but only 5.4 million will have access to RRT. It was observed, in the analysis, that from July 2013, the figures spent on RRT for the state of PB exceeded R$ 2.5 million per month, and by the end of 2013, we reached a total expenditure of R$ 32,107,67.87 used only for costs with RRT for that year, without counting the costs associated with care such as emergency hospitalizations and intensive care unit, and whose RRT is not usually charged by the UHS table but paid from outsourced contracts. The increased frequency of patients on PD and KT also has an impact on improving quality of life and reducing costs involved in the care of patients on RRT^4^.

## CONCLUSION

Secondary data from APAC allow details on the population with CKD assisted by the UHS and the assistance provided in RRT, including allowing historical monitoring of data and providing an option for local knowledge about the disease for researchers and managers, even for local where there is little or no published data about patients or care provided in RRT.
